# Genetic and Clinical Predictors of Left Atrial Thrombus: A Single Center Case-Control Study

**DOI:** 10.1177/10760296211021171

**Published:** 2021-06-29

**Authors:** Adrian Springer, Ruben Schleberger, Florian Oyen, Boris A. Hoffmann, Stephan Willems, Christian Meyer, Florian Langer, Renate B. Schnabel, Paulus Kirchhof, Reinhard Schneppenheim, Marc D. Lemoine

**Affiliations:** 1 Department of Cardiology, University Heart and Vascular Center, Hamburg, Germany; 2 Asklepios Hospital St. Georg, Hamburg, Germany; 3 Department of Pediatric Hematology and Oncology, University Medical Center Hamburg-Eppendorf, Hamburg, Germany; 4 Asklepios Hospital Harburg, Hamburg, Germany; 5 DZHK, partner Site Hamburg/Kiel/Lübeck, Germany; 6 Division of Cardiology, Cardiac Neuro- and Electrophysiology Research Consortium (cNEP), EVK, Düsseldorf, Germany; 7 Department of Oncology and Hematology, University Cancer Center Hamburg, University Medical Center Hamburg-Eppendorf, Hamburg, Germany; 8 Institute of Cardiovascular Sciences, University of Birmingham, Birmingham, UK

**Keywords:** atrial fibrillation, echocardiography, biomarkers, genetic association studies, left atrial thrombus, von willebrand factor, transesophageal echocardiography

## Abstract

Left atrial (LA) thrombus formation is the presumed origin of thromboembolic complications in patients with atrial fibrillation (AF). Beyond clinical risk factors, the factors causing formation of LA thrombi are not well known. In this case-control study, we analyzed clinical characteristics and genetic thrombophilia markers (factor V Leiden (FVL), prothrombin G20210A (FIIV), Tyr2561 variant of von Willebrand factor (VWF-V)) in 42 patients with AF and LA thrombus (LAT) and in 68 control patients with AF without LAT (CTR). Patients with LAT had more clinical conditions predisposing to stroke (mean CHA_2_DS_2_-VASc-score 3.4 ± 1.5 vs. 1.9 ± 1.4; *P* < 0.001), a higher LA volume (96 ± 32 vs. 76 ± 21 ml, *P* = 0.002) and lower LA appendage emptying velocity (0.21 ± 0.11vs. 0.43 ± 0.19 m/s, *P* < 0.001). Prevalence of FVL, FIIV and VWF-V mutations was not different, but in the subgroup of patients <65 years (y) there was a tendency for a higher incidence of VWF-V with a prevalence of 27% (LAT <65 y) vs. 7% (CTR <65 y, *P* = 0.066). These findings warrant further investigation of the VWF-V as a risk factor for LA thrombogenesis in younger patients.

## Introduction

Atrial fibrillation (AF) is the most common sustained arrhythmia in the general population and associated with a highly elevated risk of stroke and systemic embolism.^1^ Oral anticoagulation therapy (OAC) prevents the majority of ischemic strokes in patients with AF, but there is a residual stroke rate of ca 1%/year.^2^ Many thrombo-embolic events are believed to start with thrombus formation in the left atrium (LA), often in its appendage. LA thrombus formation is driven by slow blood flow, thrombogenic factors in the atrial endothelium and in the blood (Virchow´s triad).^3^ The detection of LA thrombi in patients requires transoesophageal echocardiography. Based on observational data from transesophageal echocardiograms, the prevalence of LA thrombus in anticoagulated patients with AF ranges from 0%^4^ to 6.4%,^5^ with most studies finding LA thrombus with an incidence between 0.7% and 1.9%^5–7^ (Table 1).

**Table 1.** Overview of Studies Focusing on the Relationship Between Thrombophilic Gene Variants (Factor V Leiden [FVL], Prothrombin G20210A Variant [FIIV]) and Left Atrial Thrombogenesis (LAT: Left Atrial Thrombus), Systemic Embolism and Stroke.^8–12^

Study	Group		Prevalence of		p-Value	
		n =	FVL^D^	FIIV^D^	FVL^D^	FIIV^D^
present study	LAT	42	7%	2%	0.383	1.000
	Control	68	3%	3%		
Gökce et al, 2003^A^	LAT	37	8.1%	na	ns	na
	Control	68	8.8%	na		
Go et al, 2003^A^	Embolism	137	5.8%	na	0.36	na
	Control	214	3.7%	na		
Pengo et al, 2002 ^A^	Embolism	71	7%	12.7%	ns	<0.05
	Control	142	4.2%	4.2%		
Poli et al. 2003^A^	Embolism	200	7%	1.2%	ns	ns
	Control	136	7.3%	2.9%		
Margaglione et al, 1998^B^	Stroke <50 y	202	14.9%	5%	0.008	ns
	Healthy controls	1036	4.2%	4.2%		

^A^ Collective with Atrial Fibrillation, ^B^ Stroke <50 years, ^D^ heterozygous (na: not Assessed, ns: non-significant).

In addition to clinical conditions such as age and concomitant cardiovascular diseases, quality of anticoagulation, left atrial size and function, inherited factors could contribute to LA thrombus formation. Since predisposition for LA thombus through prothrombotic genetic variants is not established yet for atrial fibrillation, we studied gene variants known for their influence on venous thromboembolism and coronay artery disease. The factor V gene variant Leiden (FVL) and the prothrombin (factor II) gene variant G20210A (FIIV) are known risk factors for the development of venous thromboembolism^13,14^ and both have been studied regarding their effect on left atrial thrombogenesis in atrial fibrillation showing heterogeneous results (see Table 1). The recently published Phe2561Tyr variant in the von Willebrand factor (VWF) gene is a gain-of-function variant and has been proven to be an independent risk factor for early and repeated myocardial infarction in women.^15–18^ We therefore assessed clinical characteristics and 3 known prothrombotic mutations in factor V, prothrombin (factor II), and von Willebrand factor in a cohort of anticoagulated patients with LA thrombus (LAT-group) and a control group without LA thrombus.

## Methods

### Study Population

In this case-control study, we consecutively identified 42 patients between 2014 and 2017 at the university heart and vascular center Hamburg with AF and LA thrombus (acute or antecedent, LAT-group) and compared these to 68 controls with AF but without LA thrombus (control). Aiming to perform a subgroup analysis we divided our studied collective into 2 age categories (<65 y; ≥65 y). The cut-off at 65 years was chosen following the lower age category of the CHA_2_DS_2_-VASc-Score. We excluded patients with active neoplastic disease, high grade mitral valve insufficiency and mitral stenosis. All participants provided written informed consent. The study was approved by the local Ethics Committee Ärztekammer Hamburg (PV5705) and is in accordance with the ethical standards laid down in the 1964 Declaration of Helsinki and its later amendments.

### Patient and Public Involvement

Patients and the public were not involved in the design, recruitment nor conduct of the study.

### Identification of Genetic Variants

DNA was isolated from peripheral blood cells using the QIAmp DNA Blood Mini Kit (QIAGEN, Hilden, Germany) and subsequently amplified by standardized multiplex polymerase chain reaction (PCR) on the Thermalcycler (BIOMETRA, Göttingen, Germany). The Tyr2561 VWF-variant (VWF-V) fragment was identified using the 3130 Genetic Analyser (LIFE TECHNOLOGIES, Carlsbad, USA). In addition to the VWF-V we also tested for FVL and FIIV using the same multiplex PCR and fragment analysis.

### Statistical Analysis

Statistical analysis was carried out using SPSS Statistics Version 22®. For sample comparison we used the 2-sample t-test for continuous variables and fisher’s exact test for binary and categorical variables. Statistical significance was assumed at a p-value of <0.05. The univariate analysis was performed using binary logistic regression. GraphPad Prism 6® (GraphPad Software, San Diego, United States of America) was used to create the illustrating artwork.

## Results

### Baseline Characterization and Echocardiographic Investigation

Patients with LA thrombus were older (69.0 ± 9.1 vs. 63.4 ± 8.9 y; *P* = 0.002) and had more cardiovascular comorbidities, reflected in a higher CHA_2_DS_2_-VASc-Score (3.4 ± 1.5 vs. 1.9 ± 1.4; *P* < 0.001; Table 2). Patients with previous LA thrombus had a lower left ventricular ejection fraction than in control (LVEF <50%:12/34 (52.4%) vs. 5/61 (7.4%); *P* < 0.001), as well as a higher LA-volume (95.9 ± 32.2 vs. 75.6 ± 20.7 ml; *P* = 0.002) and lower LA appendage (LAA) emptying velocity (0.21 ± 0.11 vs. 0.43 ± 0.19 m/s; *P* < 0.001; Table 2) measured during atrial fibrillation.

**Table 2.** Baseline Characteristics and Comparison of Patient Collectives With Atrial Fibrillation and Left Atrial (LA) Thrombus and Without LAT (Control).

	LA thrombus n = 42	Control n = 68	p-value
CHA_2_DS_2_-VASc-Score	3.4 ± 1.5 (n = 41)	1.9 ± 1.4 (n = 63)	0.001
LVEF <50%	12/34 (52.4%)	5/61 (7.4%)	<0.001
Coronary artery disease	15/42 (35.7%)	6/62 (8.8%)	0.002
Embolic complications	9/41 (21.4%)	9/63 (13.2%)	0.427
Hypertension	33/42 (78.6%)	40/63 (58.8%)	0.131
Age (y)	69.0 ± 9.1 (n = 42)	63.4 ± 8.9 (n = 68)	0.002
Diabetes	10/42 (23.8%)	6/63 (8.8%)	0.056
Female sex	12/42 (28.6%)	15/68 (22.1%)	0.497
LAA emptying velocity (m/s)	0.21 ± 0.11 (n = 40)	0.43 ± 0.19 (n = 48)	<0.001
LA-volume (ml)	95.9 ± 32.2 (n = 40)	75.6 ± 20.7 (n = 28)	0.002

LVEF, left ventricular ejection fraction; LAA, left atrial appendage.

### Anticoagulation

**Figure 1.**
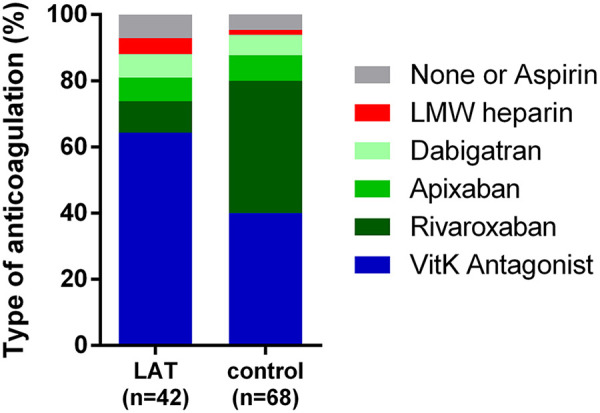
Distribution of the used anticoagulation and antiplatelet therapy: VKA (vitamin K antagonist), LMW heparin (low-molecular-weight heparin in therapeutic dosage).

Almost all patients were on anticoagulation at the time of LA thrombus detection (LAT 39/42 (93%), controls 62/65 (96%), *P* = 0.440). Direct oral anticoagulant therapy (DOAC) was used more often in controls (35/65 (54%) than in LAT-group (10/42 (24%); *P* = 0.003; Figure 1).

### Genetic Analysis

The prevalence of FVL and FIIV were not significantly different throughout the study population (Table 3). These results are similar to the expected heterozygous allele frequencies in the general population as described in the literature.^19^ The prevalence of the heterozygous VWF-V was not different in the overall comparison of the LAT-group and controls (4/42 [9.5%] vs. 9/64 [14.1%]; *P* = 0.559; Table 3). Testing for the Hardy Weinberg equilibrium we were able to estimate the expected prevalence of homozygous wildtype (77%), heterozygous Tyr2561 (21%), homozygous Tyr2561 (2%) and proved no significant difference to the observed distribution (X^2^-Test: *P* = 0.99). We did not find any homozygous Tyr2561 VWF in the studied collective. In the subgroup analysis of young patients with LA thrombus (<65 y) we found a tendency for a higher prevalence for patients with heterozygous VWF-V (Table 3). In patients with LA thrombus prevalence of VFW-V showed no correlation with an elevated VWF: RCo/VWF: Ag ratio (see table S1).^20^

**Table 3.** Prevalence of Genetic Thrombophilia Markers in Patients With Atrial Fibrillation and With (LAT) or Without (Control) Left Atrial Thrombus.^a^

	LAT	Control	*P*-value
Factor V Leiden	3/42 (7.1%)	2/64 (3.1%)	0.383
Prothrombin G20210A	1/42 (2.4%)	2/64 (3.1%)	1.000
Tyr2561 von Willebrand factor	4/42 (9.5%)	9/64 (14.1%)	0.559
Tyr2561 von Willebrand factor (<65 years)	4/15 (27%)	3/36 (7%)	0.066

^a^ Significance calculated with Fisher’s exact test.

### Predictors of LA Thrombus

**Figure 2.**
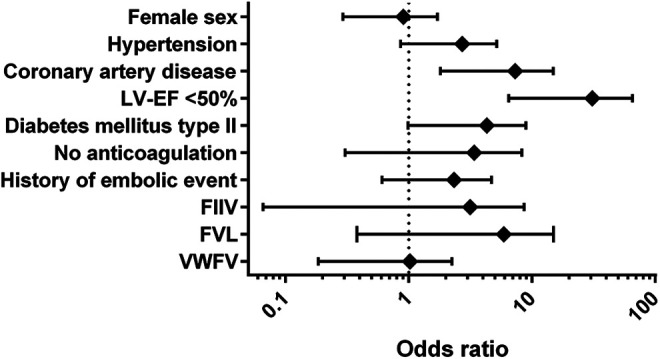
Forest plot on logarithmic scale of the Odds ratios calculated in a univariate analysis using binary logistic regression of potential categorical risk factors for LA thrombus in patients with persistent atrial fibrillation.

**Figure 3.**
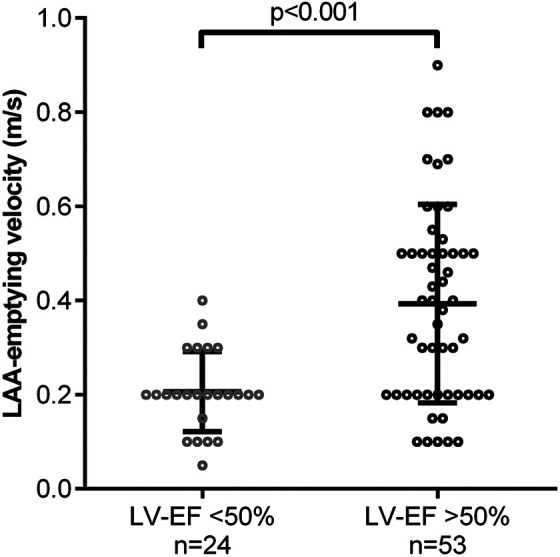
Emptying velocity of the left atrial appendage (LAA; m/s) in relation to left ventricular ejection fraction (LVEF) impairment; data presented as value, mean and standard deviation; significance calculated by unpaired t-test.

Univariate analysis including the categorical risk factors from the CHA_2_DS_2_-VASc-Score revealed reduced LVEF (OR 20.5; 95%CI: 6.5-61.1) and coronary artery disease (CAD, OR 5.2; 95%CI: 1.8-14.9; Figure 2) as independent risk factors of LA thrombus. To illustrate the apparent coincidence of reduced LVEF and reduced LAA-emptying velocity, we plotted the LAA-emptying velocities for patients with LVEF impairment (0.21 ± 0.02 m/s) vs. patients without LVEF impairment (0.39 ± 0.03 m/s, *P* < 0.001; Figure 3). The subgroup analysis of patients with LAT <65 y revealed a higher prevalence of reduced LVEF compared to older patients with LAT (LAT ≥65 y), as well as a higher prevalence of CAD in the older collective, while other known risk factors and the use of DOAC did not differ (see Table S2).

## Discussion

In this study, we found that left atrial size and function, estimated by echocardiography, is disturbed in patients with left atrial thrombus developing on anticoagulation. These factors enhance prediction of left atrial thrombi, while genetically determined prothrombotic conditions appear to play a minor role.

The association between reduced LAA emptying velocity, higher LA volume and the development of LA thrombus might be due to the pathophysiological consequences of these hemodynamic changes. Analogically to Virchow’s triad reduction in blood flow, as seen in reduced LAA emptying velocity, may lead to blood stasis facilitating thrombus formation. LA dilatation by volume increase on the other hand is associated with structural changes of the endocardial extracellular matrix, endocardial dysfunction and damage, as well as higher concentrations of prothrombotic blood constituents which are all factors that are known to predispose for thrombogenesis.^21–23^ This underlines the relevance of cardiac imaging, particularly transesophageal echocardiography, as a method for screening and risk stratification for LA thrombus.

The factor V gene variant Leiden (FVL) and the prothrombin (factor II) gene variant G20210A (FIIV) are known risk factors for the development of venous thromboembolism.^13,14^ These prothrombotic genetic variants have a similar prevalence in the general population (3-8% for FVL and 1-3% for FIIV)^19^ compared to patient cohorts with thromboembolic events (5.8-8.1% for FVL and 1.2-2.2% for FIIV)^13,14,8–11^ and both have been studied regarding their effect on LA thrombogenesis in AF showing heterogeneous results (see Table 1). In one study, the FIIV was found with a higher prevalence of this genetic variant in a cohort of patients with AF and systemic embolism compared to controls with AF without history of systemic embolism (12.7% vs. 4%, OR 3.3; 95%-CI: 1.1-9.6).^8^ Several following studies on patient cohorts with AF were not able to confirm this association.^10–12^ In line with these studies, we found no difference in the prevalence FVL and FIIV regarding the development of LA thrombus in AF.

The von VWF is a multimeric plasma glycoprotein with an important role in haemostasis and thrombus formation.^24^ High VWF plasma levels are an established independent risk factor for LA thrombus and stroke in patients with AF, but most of the previous studies have mainly focused on quantitative abnormalities in VWF expression.^19^ Qualitative changes in VWF activity in relation to LA thrombus and stroke have not been assessed before. The recently published Phe2561Tyr variant in the VWF gene is a gain-of-function variant and has been proven to be an independent risk factor for early and repeated myocardial infarction in women.^15–17^ Structural and functional analysis indicate that the VWF-V is more susceptible to shear stress induced elongation, which is mandatory for its proper function and therefore conveys a prothrombotic state in carriers of this genetic variant.^17,18^

The potential association of the VWF-V to LA thrombogenesis in AF was investigated for the first time and the prevalence of the VWF-V in LAT-group and controls was not significantly different (OR: 0.64; 95% CI: 0.19-2.24) and similar to the expected heterozygous allele frequency (9.8% in a cohort of neonates).^15^ A possible explanation might be found by looking at the underlying pathophysiology of thrombus formation. LA thrombogenesis is mainly based on blood stasis, with reduction of LAA-emptying velocity and LA dilatation being important risk factors.^19^ The VWF-V on the other hand conveys its prothrombotic effect through an increased shear stress susceptibility.^17,18^ Therefore, the prothrombotic relevance might be stronger associated to conditions with increased shear stress on the blood constituents as apparent in elevated blood flow velocity (e.g. on atherosclerotic plaques in CAD).^25^

Interestingly, we found a tendency for a higher prevalence of the VWF-V in the subgroup with AF and diagnosis of LA thrombus <65 years. These findings are consistent with a previous study performed by Treder^15^ and Schneppenheim et al^17^ who tested for the VWF-V in a large cohort of 2070 patients with different stages of CAD and myocardial infarction and demonstrated a significantly higher prevalence of the VWF-V in a subgroup of 13 female patients with ≥2 myocardial infarctions (MI) <55 years (30.8% vs. 7%, p = 0.043) without finding significant differences in the whole study population. The higher prevalence of VWF-V in the subgroup of young patients with thrombotic event (in our case LA thrombus) might be explained by the minor prevalence of cardiovascular risk factors (hypertension, diabetes, congestive heart failure, vascular disease) in younger patients and a potentially increasing influence of genetic factors.^26^ Comparing older and younger patients with LA thrombus we found overall similar prevalences of known risk factors, except for LVEF impairment, which was more common in the younger group (Supplement Table 2) and as previously described, seems to be a major risk factor for the development of LAT. The lower prevalence of the VWF-V in older patients might also be explained by the suspected higher risk for embolic complications and thus limited life expectancy in carriers of this variant. Further longitudinal studies closely monitoring this patient collective could confirm or refute this theory.

Specific and individualized therapy for patients with LA thrombus and AF is crucial to balance between thrombotic and bleeding complications. Due to the lack of large study cohorts, ESC guidelines only offer advice regarding the acute management of LA thrombus. In practice, pharmacological therapy aims for early initiation of OAC, or, if OAC is already established with sufficient patient compliance, OAC might be modified as an individualized approach to help reducing LA thrombus size. Potential modifying strategies are e.g. elevating INR target range, changing from DOAC to VKA or vice versa.

A specific therapy might become available for pathological VWF activity. A recent study focusing on stroke outcomes and prophylactic options in patients with elevated VWF levels showed positive results using the GPIb-VWF blocker caplacizumab to reduce infarct size in pre-clinical studies. The applicability in a human cohort as well as possible bleeding complications and contraindications remain to be tested, nonetheless a tendency to individualization of therapy is already apparent. Identifying carriers of the VWF-V might gain importance in this context to screen for patients who would profit from such a direct therapeutic approach.

Furthermore, the gathered echocardiographic data suggests a strong association between the reduction of LVEF and the LAA-emptying velocity. Individuals with apparent reduction of LVEF showed a considerably lower LAA-emptying velocity, which is known to predispose for LA thrombus development.^27^ This might explain the results of the univariate analysis identifying the reduction of LVEF as the major risk factor for LA thrombogenesis in the study population. Although the intracardiac hemodynamic relations in AF are far more complex, this connection could account for part of the increased risk to develop LA thrombus and thromboembolic events conveyed by the reduction of LVEF. Transferred to clinical practice, patients with atrial fibrillation and reduced LVEF as the only risk factor of the CHA_2_DS_2_-VASc-Score might be rather considered for OAC than patients with less impacting single risk factors (e.g. female sex category, vascular disease).^28^

Interestingly, the distribution of the antithrombotic compounds in the LAT-group compared to controls seems to suggest an overall benefit of DOAC compared with VKA in preventing LA thrombus. However, due to the individualized decision, on which anticoagulant agent is used in which patient, considering possible contraindications and surrounding circumstances a definite statement cannot be made as to which agents are more efficient in preventing LA thrombus. Nonetheless, many studies demonstrated the difficulties of VKA therapy with a time in therapeutic range of 55-70%, which might explain a slightly higher thromboembolic risk during VKA therapy.^2^

### Study Limitations

The main limitation of this study is the limited cohort size. To perform a conclusive genetic study very large cohort sizes are needed, to reach statistically significant results. This can be problematic when studying comparatively rare pathologies, such as LA thrombus in this case. With an estimated homozygosity us genotype frequency for VWF-V of 2%, our patient sample was too small to expect a homozygous VWF-V.^15^ Future large scale cohort studies should be able to solve this problem by generating sufficient patient numbers, as well as prospectively gathering information on possible impairment of life expectancy due to the VWF-V.

## Conclusion

In conclusion, this study found that age, reduced LVEF and LAA emptying velocity, as well as a higher LA Volume and CHA_2_DS_2_-VASc-Score are associated with formation of LA thrombus on anticoagulation in patents with AF, whereas genetic risk factors (VWF-V, FVL and FIIV) were not associated with their formation in this cohort. Further studies of left atrial size and function, and potentially of genetic markers in young patients, are warranted to confirm these findings.

## Supplemental Material

Supplemental Material, sj-docx-1-cat-10.1177_10760296211021171 - Genetic and Clinical Predictors of Left Atrial Thrombus: A Single Center Case-Control Study Click here for additional data file. Supplemental Material, sj-docx-1-cat-10.1177_10760296211021171 for Genetic and Clinical Predictors of Left Atrial Thrombus: A Single Center Case-Control Study by Adrian Springer, Ruben Schleberger, Florian Oyen, Boris A. Hoffmann, Stephan Willems, Christian Meyer, Florian Langer, Renate B. Schnabel, Paulus Kirchhof, Reinhard Schneppenheim and Marc D. Lemoine in Clinical and Applied Thrombosis/Hemostasis
