# Supplementary material for: Genetic and Clinical Predictors of Left Atrial Thrombus: A Single Center Case-Control Study
Source: Clin Appl Thromb Hemost. 2021 Jun 29;27:10760296211021171. doi: 10.1177/10760296211021171 (PMC8246465; doi:10.1177/10760296211021171)
Supplement: Supplemental Material, sj-docx-1-cat-10.1177_10760296211021171 - Genetic and Clinical Predictors of Left Atrial Thrombus: A Single Center Case-Control Study [file sj-docx-1-cat-10.1177_10760296211021171.docx]

**Table S1:** Comparison of VWF activity index (VWF:Act) measured by VWF ristocetin cofactor activity divided by VWF antigen (VWF:RCo/VWF:Ag) in patients with LAT with (VWF +) and without (VWF -) heterozygous VWF-V

|  | VWF + (n=3) | VWF - (n=32) | p-value | ref. range^1^ |
| --- | --- | --- | --- | --- |
| VWF:Rco (%) | 127,5±4 | 153±55 | 0.441 | 61-179% |
| VWF:Ag (%) | 172±34 | 198±97 | 0.656 | 50-160% |
| VWF:RCo/VWF:Ag (VWF:Act) | 0.78±0.19 | 0.84±0.2 | 0.615 | 0.6-2.0 |

1. Budde U, Drewke E, Will K, Schneppenheim R. Diagnostic standards of von Willebrand disease. *Hamostaseologie*. 2004;24(1):12-26. doi:10.1055/s-0037-1619602

**Table S2**: Baseline characteristics and comparison of patient collectives with atrial fibrillation and left atrial thrombus (LAT) divided in age-categories <65 and ≥65 years.

|  | LAT<65y (n=15) | LAT≥65y (n=27) | p-value |
| --- | --- | --- | --- |
| CHA_2_DS_2_-VASc-Score | 2.6±0.99 (n=15) | 3.9±1.59 (n=26) | 0.083 |
|  |  |  |  |
| LVEF <50% | 10/13 (77%) | 11/20 (55%) | 0.015 |
| Coronary artery disease | 2/15 (13%) | 13/27 (48%) | <0.005 |
| Embolic complications | 4/15 (27%) | 5/27 (19%) | 0.299 |
| Hypertension | 11/15 (73%) | 22/27 (81%) | 0.249 |
| Diabetes | 4/15 (27%) | 6/27 (22%) | 0.540 |
| Female sex | 5/15 (33%) | 7/27 (26%) | 0.350 |
| use of DOAC | 3/15 (20%) | 7/27 (26%) | 0.163 |
| LAA emptying velocity (m/s) | 0.21±0.14 (n=15) | 0.21±0.09 (n=25) | 0.253 |
| LA-volume (ml) | 103±41 (n=15) | 92±26 (n=25) | 0.331 |
